# Women’s experiences of a telemedicine abortion service (up to 12 weeks) implemented during the coronavirus (COVID‐19) pandemic: a qualitative evaluation

**DOI:** 10.1111/1471-0528.16813

**Published:** 2021-07-27

**Authors:** N Boydell, JJ Reynolds‐Wright, ST Cameron, J Harden

**Affiliations:** ^1^ Usher Institute University of Edinburgh Edinburgh UK; ^2^ NHS Lothian Edinburgh UK; ^3^ MRC Centre for Reproductive Healthcare University of Edinburgh Edinburgh UK

**Keywords:** Health services research, qualitative research, teleconsultation/telehealth, termination of pregnancy, termination of pregnancy: medical, women’s experiences

## Abstract

**Objective:**

To explore the experiences of women in Scotland who accessed medical abortion at home up to 12 weeks’ gestation, delivered via a telemedicine abortion service implemented in response to the coronavirus (COVID‐19) pandemic, to identify areas for improvement and inform service provision.

**Design:**

Qualitative interview study.

**Setting:**

Abortion service in one National Health Service health board in Scotland.

**Population or sample:**

Twenty women who accessed telemedicine abortion services and self‐administered mifepristone and misoprostol at home up to 12 weeks’ gestation.

**Methods:**

Thematic analysis of semi‐structured qualitative interviews, informed by the Framework analytic approach.

**Main outcome measures:**

Women’s experiences of accessing telemedicine for medical abortion at home, specifically: acceptability of the telephone consultation and remote support; views on no pre‐abortion ultrasound scan; and self‐administration of abortion medications at home.

**Results:**

Novel study findings were three‐fold: (1) participants valued the option of accessing abortion care via telemedicine and emphasised the benefits of providing a choice of telephone and in‐person consultation to suit those with different life circumstances; (2) the quality of abortion care was enhanced by the telemedicine service in relation to access, comfort and flexibility, and ongoing telephone support; (3) participants described being comfortable with, and in some cases a preference for, not having an ultrasound scan.

**Conclusions:**

This research demonstrates support for the continuation of telemedicine abortion services beyond the temporary arrangements in place during COVID‐19, and lends weight to the argument that offering the option of telemedicine abortion care can enable women to access this essential health service.

**Tweetable abstract:**

#Telemedicine provision of medical #abortion at home up to 12 weeks’ gestation is acceptable and highly valued by #women #Research #SRHR @nbw80 @doctorjjrw @jeniharden @cameronsharon @mrc_crh @edinuniusher.

## Tweetable abstract

#Telemedicine provision of medical #abortion at home up to 12 weeks’ gestation is acceptable and highly valued by #women #Research #SRHR @nbw80 @doctorjjrw @jeniharden @cameronsharon @mrc_crh @edinuniusher.

This article includes Author Insights, a video abstract available at: https://vimeo.com/bjog/authorinsights16813

## Graphical abstract

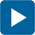

## Introduction

Prior to the coronavirus (COVID‐19) pandemic, women and pregnant people in Great Britain who chose medical abortion (<10 weeks’ gestation) at home, were required to make an in‐person visit to an abortion service for assessment; this usually involved a routine ultrasound scan to confirm gestation.^1^ Mifepristone was received in the clinic; however, the second part of medical abortion treatment (misoprostol) could be self‐administered at home.^1^, ^2^

In response to COVID‐19, the Departments of Health in England, Wales and Scotland introduced legislation allowing women to self‐administer mifepristone in their place of residence, in addition to misoprostol.^3^, ^4^, ^5^, ^6^ Coupled with clinical guidance from the Royal College of Obstetricians and Gynaecologists^7^ that encouraged remote consultations and limited the need for routine pre‐abortion ultrasound, telemedicine abortion services were implemented. In Scotland, home administration of mifepristone and misoprostol is available up to 12 weeks’ gestation; in England and Wales, due to differences in legislation, it is only available before 10 weeks’ gestation.^3^

One of Scotland’s largest abortion services is in Lothian (Edinburgh and surrounding region) and is provided by the National Health Service (NHS) free at the point of access.^8^ Those seeking abortion can self‐refer (by telephone) or can be referred by a clinician, such as a GP.^9^ The NHS Lothian telemedicine model involves a telephone consultation with a clinician, during which information about the abortion process and post‐abortion contraception is provided and verbal consent for the procedure is obtained. This is supplemented by written information (in the treatment pack) and audiovisual information available via a dedicated website.^10^ If the person seeking abortion had a certain last menstrual period under 12 weeks previously and is without signs, symptoms or significant risk factors of ectopic pregnancy, an ultrasound is not required.^11^ For those choosing medical abortion at home, the medications are later collected or delivered to them.^12^ Details of the treatment regimen are available as supplementary material (Table S1). Some may need, or prefer, to attend in‐person appointments within clinics, for example, where they are insecurely housed or where there are safeguarding issues. In such circumstances in‐person consultations continue to be available.

Previous research demonstrates that direct‐to‐patient and clinic‐supported telemedicine abortion services are valued by women in contexts where there is restricted access to free and/or legal abortion.^13^, ^14^, ^15^, ^16^, ^17^ However, there are limited data on the acceptability of clinic‐supported telemedicine in settings similar to Scotland, where abortion is legal, easy to access and available free at the point of access.^18^, ^19^ As such, the study objective was to explore (i) the acceptability of the telemedicine abortion consultation, and clinical support, to those seeking abortion in Scotland; (ii) views on not receiving a pre‐abortion ultrasound scan; and (iii) experiences of self‐administration of both mifepristone and misoprostol at home. Given that the telemedicine service was implemented in response to COVID‐19, we also sought to investigate how this may have impacted experiences of abortion. The findings will contribute to abortion policy and practice following COVID‐19.

## Material and methods

### Research design

We designed a qualitative evaluation utilising individual, semi‐structured interviews, to explore the experiences of women that accessed medical abortion (via the NHS Lothian telemedicine abortion service), with home self‐administration of mifepristone and misoprostol.

### Sampling

Our aim was to recruit a sample of women accessing the NHS Lothian telemedicine abortion service during the period of the qualitative service evaluation. A purposive sampling approach was guided by considerations around study resources and duration.^20^, ^21^ Inclusion criteria included: obtaining medical abortion, under Ground C of the 1967 Abortion Act^22^ (i.e. not for medical conditions or fetal abnormality); being eligible for medical abortion at home; aged 18 or over; and fluent in speaking, reading and understanding English. Data collection ended when no new issues arose from further data collected (‘data saturation’).^20^

### Recruitment

Recruitment took place between May 2020 and July 2020 via two routes. First, a participant information sheet (PIS) was included with the medication pack for those accessing medical abortion at home, inviting them to contact the research team to discuss participation. Secondly, during planned follow‐up telephone consultations 10–14 days after the initial consultation and provision of abortion medications, clinicians from the abortion service mentioned the PIS, and briefly outlined the study. Where interest was expressed by a potential participant, permission was sought to share their contact details with the research team. A researcher called those who granted permission 1 week after receipt of contact information (approximately 3 weeks post‐abortion) to discuss the study and, where agreed, arrange a telephone interview. Of the 54 potential participants who agreed to be contacted by the research team, 20 agreed to an interview.

### Data collection

Individual semi‐structured telephone interviews were conducted with participants. An interview topic guide (see Appendix S1) was used to structure the discussion, covering: experiences of the telephone consultation; receiving/providing information; contraceptive counselling; views on no ultrasound scanning; access to medications; undertaking the abortion process at home; pre‐ and post‐ abortion support; views on future service delivery and the impact of COVID‐19 on their experiences.

All telephone interviews were conducted by either NB or JH, both of whom are social scientists with extensive experience of qualitative research on sexual and reproductive health. Interviews were digitally recorded (using an encrypted recorder) and typically lasted for 45 minutes. Informed consent was obtained (recorded verbally) prior to the interviews. Participants received a £15 voucher in recognition of their time.

### Data analysis

The qualitative data were analysed thematically by the team (NB, JH and JRW) using an approach informed by the Framework analytic method.^21^, ^23^, ^24^ All interviews were transcribed verbatim, pseudonymised and entered into NVIVO qualitative data analysis software (version 12, QSR International (UK) Limited The Innovation Centre, Cheshire, UK) to facilitate data management and coding, and the generation of thematic Frameworks. Transcripts were subject to repeated reading and comparison to identify recurrent issues, including those not foreseen at the study outset. A coding framework, which captured both the original research questions and emergent issues, was developed and applied to the interview data. Coded datasets were then subject to further in‐depth analysis to support the construction of themes and sub‐themes.

### Patient and public involvement

The focus of the study was patient involvement in informing future services. Patients were not directly involved in the design, recruitment for or conduct of the interviews. A summary of the research findings was offered to participants.

## Results

The final sample comprised 20 participants; sample characteristics are outlined in Table 1. Through our analysis, issues relating to telemedicine abortion were grouped into four thematic areas: (1) access to the telemedicine service; (2) experience of the telephone consultation; (3) accessing and administering abortion medications, and managing the abortion at home; and (4) views on future abortion service provision. Participants’ accounts provided limited data around the impact of COVID‐19 on their experiences of abortion; as such, we present COVID‐19‐related findings, where relevant, within themes rather than as a separate theme. Extended data extracts are presented in Tables 2, 3, 4.

**Table 1:** Characteristics of study participants (*n* = 20)

Characteristic	Total
Age (years)	
18–25	8
26–32	7
33–39	5
Highest education level attained	
Secondary school	4
School or college 6th form	1
College of further education	4
Polytechnic or University	11
Employment status	
Employed	11
Self‐employed	2
Student	6
Unemployed/looking after home	1
Ethnicity	
White British/Scottish/English/Northern Irish	17
White other	2
British Asian	1
Relationship status	
Single	2
In relationship (not co‐habiting)	5
Co‐habiting/married	11
Separated/divorced	2
Gestation (in weeks)	
<6 weeks	7
6–10 weeks	2
10–12 weeks	1
Not disclosed during interview	9
Previous pregnancy	
Yes	11
Previous abortion	
Yes	4
Contraceptive use prior to abortion	
None	11
User controlled method*	8
LARC**	1

* Reported user controlled methods of contraception: combined oral contraceptives (COCs), Progestogen‐only pills (POPs) and condoms (Male).

** Reported long acting reversible contraception (LARC) method was intrauterine contraception (IUC).

**Table 2:** Example extracts: access to the telemedicine abortion service

Extract no.	Extract
**1**	‘I called [the abortion service] straight away and they asked me if I was available I think it was the next day for a telephone call and I said yes that’s absolutely fine so it was quite quick.’ (P014; age 26–32)
**2**	‘…it (telemedicine abortion access) best for me because obviously it was over the phone and it was done quick and things like that which I wanted, whereas maybe if the virus wasn’t here then maybe obviously I would need to go in face to face and things like that’ (P004; age 26–32)
**3**	‘…it being a telephone conversation you don’t have to allow for travel time, dropping kids of somewhere so it meant that I could just say to my husband ‘this is the time that you need to be watching them for me’ and he managed to fit that in with his work just because I could tell them what time was appropriate for them to phone me back, so I could take myself away into a separate room and know that I wasn’t going to be interrupted when I was on the phone.’ (P018; age 26–32)
**4**	‘…being able to just do it from home makes it more convenient and not having to go to the clinic or anything cause I stay quite far out from town, so going into town would have been a bit of a hassle for me so it was just a lot more convenient being able to just be able to do it at home, where I’m kinda more comfortable as well.’ (P016; age 26–32)

**Table 3:** Example extracts: experiences of the telephone consultation

Extract no.	Extract
5	‘…the consultation that I had with the nurse who was so good, she was so thorough, she went over everything over and over again to the point, where I was like ‘yeah I get it’ […] I appreciated that because it was nice that she was giving me that time I think. I didn’t feel like a conveyor belt number, I felt like a person who was getting the time to ask questions and take all the information in.’ (P012; age 33–39)
6	‘I mean, they explained it all very well and they give you, like, this whole detailed thing of, like, step by step guide and stuff.’ (P019; age 26–32)
7	‘She gave me a lot of information and helped me kind of understand what I’d be going through and what my body would be going through. She gave me a lot of time to talk about, like, anything I wanted to or if I was scared about anything or if I had any questions about any of the tablets and that and she made me feel, like I said, she made me feel really comfortable, if I needed anything explained again she would say it again and just explain it in a bit more depth.’ (P003; age 18–25)
8	‘…it was really easy to talk to them on the phone, I felt like, they were really understanding and when I said that I’d been emotional they took time to talk about that which was nice […] I didn’t feel like it was impersonal and just a list of questions, I felt like I had time to talk it through.’ (P018; age 26–32)
9	‘I’m used to writing things down and actively listening when someone’s talking and I can’t see them, so it was much easier for me to do that and I could just sort of doodle stuff down on a bit of paper. It was all stuff that had been put in the information leaflet when I picked up my medication anyway, so it was all there for me but it was just, you know, certain things that I wanted to write down just as a reminder or a refresher […] you can’t really do that when you’re speaking to someone face to face I suppose, it seems that you shouldn’t really do that, you should be concentrating and listening to them, but I feel like over the phone you can get away with just writing down a little thing just to remind you.’ (P015; age 26–32)
10	‘I was quite nervous at the start cause I wasn’t really sure what she was going to say or what… I’ve never really had to go through anything like that before so I was a bit nervous about what the process involved and, like, what she was going to tell me I had to do, but like being at home, I don’t know, it made me feel a bit more comfortable and being able to talk a bit more, like, I feel like if she’d asked me questions or if I had any concerns I would’ve probably been able to say them in person, but because I was in my own space I felt more comfortable and more willing to ask questions and stuff.’ (P003; age 18–25)
11	‘I can take hours to work myself up, if I don’t talk myself out of it, which 90% of the time I do. The amount of appointments I’ve missed because I can’t go out and end up not going, cause I freak out and just can’t, then I lose my appointment’ (P007; age 33–39)
12	‘I find it hard to talk to people face to face sometimes, especially in uncomfortable situations and, I think on the phone you’re kinda a little bit separate from them cause it is just a phone call and you’ve not got someone sat in front of you, staring at you and watching you, which some people might need obviously if they prefer that, but for me it’s much easier to not be in front of someone.’ (P006; age 18–25)
13	‘…it’s much more comfortable and just in terms of confidentiality, it’s a bit more, I don’t know, I use the word secret, you don’t have to be seen to potentially be going into a clinic that you don’t want people, if you’re a professional person, you might not want somebody to see you do that. So I think it’s quite a good thing that it can be done over the telephone from the comfort of your own home.’ (P008; age 26–32)
14	‘I think it just depends whether you have, a private space to talk or whether, I don’t know, you live in a busy household and maybe your family doesn’t know. Yeah, I didn’t have that problem but that would be something I would consider if I was in that position, I would’ve rather done it face to face but because I was able to phone, and it be completely confidential sort of thing, it was really helpful.’ (P011; age 18–25)
15	‘I made the phone call in the car so that I didn’t have to come home and do it when my kids were here or anybody else in the house, so I just made the phone call in the car and just sorted it out then […] It would just be me and the two children and then my partner is here, he works Monday to Friday type thing, so he would be in in the evenings. He’s fully aware of all this though, it wasn’t that I was keeping it from him, it was just I didn’t want to have the kids in the background, you know.’ (P012; age 33–39)
16	‘I preferred not to have one [scan], like I say we had talked about it and we weighed up sort of pros and cons either to keeping, or terminating the pregnancy […] and for me to have to have an ultrasound would’ve been much more difficult because I think cause I’d only ever had that situation with the two pregnancies I’ve gone ahead with before it’s always been something, like, exciting, a positive experience, looking forward to doing that so for me to go in and do that I think it would’ve then been harder to go ahead and terminate the pregnancy. I was really relieved at not having to have one to be honest’ (P018; age 26–32)
17	‘…getting a scan it kinda makes it real, when you think of getting a scan you just automatically think of a baby and pregnancy and things like that in a happy time, whereas, not that this was my case, but if you had fell pregnant in a bad situation I think the scan could make it quite emotional, that’s just my opinion. I feel like in my early stage [pregnancy] I don’t think it would’ve been required.’ (P015; age 26–32)

**Table 4:** Example extracts: accessing abortion medications and managing the abortion process at home, and future service provision

Extract no.	Extract
18	‘I just wanted to get it done, I didn’t want to wait and again I understand that every day on waiting or anything this means that more days on my pregnancy, more risks, so I just want to get it done as fast as possible. I know that it’s next day delivery, it’s just changing by one day but because I was on my phone call since the morning I was just like ‘let’s get it, let’s get it done’‘ (P002; age 33–39)
19	‘I think I just like to be in control about going to get it and knowing that I had it rather than somebody delivering it.’ (P013; age 18–25)
20	‘I don’t think it was complicated in any way, it was really easy and, like, in the packs as well there was loads of information so if I did have, like, it was all labelled really well if that makes sense. It was just quite clear, so they all had their own individual polypocket with, like, what it was, when to use it, why you’re using it, things like that.’ (P011; age 18–25)
21	‘I think it was quite simple, you know, just written in plain English and very simple instructions, so to me it was very clear. Everything was mentioned as well, like, what can happen to you, you know, good to know, like, maybe you will not have all the side effects mentioned but it’s good to know everything I think, quite important.’ (P014; age 26–32)
22	‘I think also what helped was that the different boxes of medication, although they had their actual names on them, there’d been stickers put on the side of them saying ‘anti‐sickness’ and ‘this one for here’ and ‘antibiotics’ so that it was a bit more clear that straight away that’s what them ones were, that’s what them ones were, and that was helpful too.’ (P010; age 18–25)
23	‘You can’t really avoid anyone especially when you’re in a small flat, you’re kinda stuck where you are.’ (P006; age 18–25)
24	‘It was good that I didn’t have to go into work cause of lockdown and being able to do it at home, cause obviously with work then I’d have to push back clients and stuff, whereas with the whole lockdown I’m not working so I knew that I didn’t have that to worry about’ (P016; age 26–32)
25	‘I knew about all the things what could go wrong but again the good thing about all information what I was given, there was a mobile phone number to the nurse who I can phone any time pretty much if I have any problems or not sure about something, and that was again they said if I feel like if something go wrong I have a numbers where I can phone and I have all information where I need to go and what I need to do. So that again of course I was worried but in the same time I was feeling safe because I knew exactly what steps I need to do if something is wrong.’ (P002; age 33–39)
26	‘I think for people maybe who are a bit like me who are just a bit more… they just feel a wee bit more pressure I think it’s a great service to do it over the phone. Obviously maybe still having that option if someone does want to go in face to face or they have to, then that’s fine but I found the telephone part of it was really good and really helpful, and as I said it made me feel more relaxed and at ease rather than having to go all the way up to have a face to face conversation with someone about it. So I found it was really good and, I mean, hopefully they can keep the telephone service cause it maybe will help a lot more people to, like, maybe phone up and do it instead of just ignoring it, I don’t know, but I found it was really good and helpful. And as I said it was done quick and everything was explained just as good probably if someone was to do it face to face.’ (P004; age 26–32)
27	‘I definitely think that [telemedicine abortion] should be an option […] It was perfect for me the whole process, but that absolutely wouldn’t be right for somebody else. But no it was incredibly convenient, you know, there’s no denying that and the stress was kept to a minimal. I guess I think it doesn’t have to be a complicated process particularly if that individual’s sure, of what’s going on, so it’s I guess about that assessment on the phone and it might be more appropriate to get someone in who’s less sure of going ahead, yeah. Yeah, giving the option but also picking up where that individual’s at.’ (P005; age 33–39)

### Access to the telemedicine abortion service

Several benefits of the telemedicine service relating to access were articulated: ease of access, timeliness, and convenience and flexibility.

The process of accessing the abortion service was described as easy and straightforward. Many participants self‐referred to the service, whereas others first contacted their GP. The telemedicine service was understood as facilitating timely access to abortion, thus addressing (real and perceived) time pressures. All participants received an offer of a telephone consultation within 24–48 hours, with several receiving a consultation the same day they made initial contact. The short waiting time helped to address anxiety about the process and about being able to obtain a medical abortion quickly.

I called [the abortion service] straight away and they asked me if I was available I think it was the next day for a telephone call […] so it was quite quick. (P014; age 26–32)

The convenience of receiving a telephone consultation was emphasised; particularly, that it offered flexibility in both timing and location, and reduced the need for travel. This enabled participants to make arrangements that aligned with their personal circumstances, including childcare and working patterns. (Table 2).

…being able to just do it from home makes it more convenient and not having to go to the clinic or anything […] being able to just be able to do it at home, where I’m kinda more comfortable as well. (P016; age 26–32)

### Experiences of the telephone consultation

All participants described their experience of the telephone consultation as positive, and as good as in‐person care, with regard to communication, building rapport and information sharing. Participants described feeling ‘cared for’ (during and after) the telephone consultation. The sensitive, caring and holistic approach of staff was emphasised, specifically the attention given to their personal circumstances, attunement to their emotional state, and the support and reassurance offered.

I didn’t feel like a conveyor belt number, I felt like a person who was getting the time to ask questions and take all the information in. (P012; age 33–39)

The ‘step‐by‐step’ approach staff took in explaining the process was valued; participants emphasised that information was presented clearly, without use of medicalised language, and that ample time was provided to ask questions. Some participants noted that the telephone consultation allowed them to write notes, without concerns about being observed.

…they explained it all very well and they give you, like, this whole detailed thing of, like, step by step guide and stuff. (P019; age 26–32)

The benefits of the consultation taking place at ‘home’, a familiar space where many participants felt more comfortable and ‘at ease’, were highlighted. Some participants described sitting in bed with a coffee, able to wear pyjamas or comfortable clothes. The ability to choose the location for the consultation contributed to feelings of comfort and control over the process, enabling participants to feel more ‘open’ and raise concerns during the consultation. The telemedicine service was particularly welcomed by those who disclosed experience of anxiety and depression, and who found in‐person attendance at appointments challenging.

…I feel like if she’d asked me questions or if I had any concerns I would’ve probably been able to say them in person, but because I was in my own space I felt more comfortable and more willing to ask questions. (P003; age 18–25)

Conducting the consultation by telephone alleviated concerns about ‘visibility’ and being judged by others, or about breaches of anonymity and confidentiality. For some participants the issue of visibility was explicitly linked to fear of judgment and abortion stigma, which consulting by telephone went some way to address.

Nevertheless, some participants noted privacy challenges with the telephone consultation. Finding ‘private’ space where they would not be overheard was especially important for those who did not want to disclose an abortion to family members, flatmates, parents or other household members. COVID‐19 ‘lockdown’ conditions at times further complicated this, particularly for participants who were unable to be in their preferred location or who had to make additional arrangements.

Scanning and gestational stage were discussed during interviews. A few participants reported having received a scan either because they were unsure of the date of their last menstrual period or because they had already accessed the Early Pregnancy Unit due to suspected ectopic pregnancy. Of those who had not received a scan, most described being comfortable with, and preference for, not receiving a scan. For some, this related to their certainty about gestational stage, based on use of period tracker apps or personal records; others were reassured by assessment during the telephone consultation. The elimination of routine ultrasound scanning was welcomed by those who associated scans with positive experiences of (desired) pregnancy, and were anxious about the prospect of being required to undergo this during the consultation (Table 3).

…getting a scan it kinda makes it real, when you think of getting a scan you just automatically think of a baby and pregnancy and things like that in a happy time. (P015; age 26–32)

### Accessing and administering abortion medications, and managing the abortion at home

Participants were given the option of collecting medications from the service or delivery via courier; most opted to collect. Collection of medication was primarily described as enabling rapid access, to ‘get it done’; others noted that collection provided a sense of ‘control’ over the process. Those who accessed medication by courier emphasised the convenience of this but noted that some may have concerns around delivery timing and the potential for packages to be identified as abortion medication.

I think I just like to be in control about going to get it and knowing that I had it rather than somebody delivering it. (P013; age 18–25)

No problems in following the instructions or administering mifepristone or misoprostol were reported. Although participants noted that there was a large amount of written information in the treatment pack, the instructions were typically described as clear, simple and aligning well with the explanation provided during the teleconsultation. The ‘non‐medicalised’ language used to label medications was also valued.

…it was really easy and, like, in the packs as well there was loads of information […] it was all labelled really well if that makes sense. It was just quite clear, so they all had their own individual polypocket with, like, what it was, when to use it, why you’re using it. (P011; age 18–25)

Experiences of the abortion process and passing the pregnancy were largely in line with our previous, pre‐pandemic, research on medical abortion at home^2^; participants described benefits associated with being in the comfort of their own home and having control over timing. Some experienced challenges associated with the availability of their preferred supporter or location for the abortion; in some cases this was directly related to COVID‐19 ‘lockdown’ conditions. For others, the ‘lockdown’ made the process easier, as their preferred supporter was more easily available and there was no pressure to explain absence from work and/or social activities, or the need to be at home.

Participants reported feeling secure and confident that they could access support from the abortion service if required, linking this explicitly to the caring approach of clinicians during the consultation. Some reported accessing telephone or text support for reassurance around timing, pain and bleeding. Where concerns around bleeding required hospital follow‐up, support from abortion service staff in navigating this process was described as helping to alleviate anxiety (Table 4).

I was feeling safe because I knew exactly what steps I need to do if something is wrong. (P002; age 33–39)

### Future service provision

Across participants’ accounts there was unanimity that, if possible, a telemedicine abortion service should continue to be offered. A few participants had previous experience of home use of misoprostol and expressed a preference for the option of telemedicine, primarily because of convenience. In addition to the benefits of telemedicine provision already highlighted, some suggested that it could help to normalise abortion care for others. Almost all suggested that offering a choice of telephone and in‐person consultation would help meet the needs of those with different life circumstances.

…hopefully they can keep the telephone service cause it maybe will help a lot more people to, like, maybe phone up and do it instead of just ignoring it, I don’t know, but I found it was really good and helpful. (P004; age 26–32)

When asked hypothetically about the option of having a video consultation, participants reported a preference for a telephone consultation. For some, this was linked to the issue of visibility described earlier; not wanting to be visible to those providing the service.

## Discussion

### Main findings

Novel findings of our study were three‐fold. First, in the context of a country with legal access to abortion provided as part of regular healthcare, participants valued the telemedicine abortion service as an option. Many participants highlighted that it would be ideal to offer the choice of telephone and in‐person consultation to ensure that there were options to suit those with different life circumstances. This supports research in other countries where access to abortion is restricted, and highlights that the option of telemedicine abortion is acceptable in a range of contexts.^13^, ^17^ Secondly, participants typically described being comfortable with, and in many cases a preference for, not having an ultrasound scan. Thirdly, the quality of participants’ abortion care was facilitated by the telemedicine service in relation to access, comfort and flexibility, and support.

### Strengths and limitations

The rich qualitative data generated have enabled exploration of women’s experiences of accessing telemedicine abortion (introduced during the COVID‐19 pandemic), self‐administering both mifepristone and misoprostol, and self‐managing the abortion process at home; areas yet unexplored in the literature on abortion in the UK.

All participants were recruited from a single setting during the period of the first COVID‐19 ‘lockdown’ restrictions in Scotland, immediately following implementation of the service. As such, the findings may not be reflective of service users’ experiences, as the service adapts dynamically to updated guidance on service provision and the ‘post‐lockdown’ needs of those accessing the service. Women experiencing challenges in accessing abortion care, for example in the context of an abusive relationship or difficult ‘home’ circumstances, are less likely to have opted‐in to the research, therefore we may not have captured the diversity of such experiences.

It is notable that participants were highly positive about their experience of the NHS Lothian telemedicine service. There is a body of literature that suggests a tendency for recipients of healthcare to evaluate the care they receive positively, and this may have played a role here.^25^ However, NHS Lothian is active in research on abortion care, and service development and improvement, informing good practice within the service. Further research in other contexts would help ‘unpack’ factors that underpin positive experiences of telemedicine abortion provision.

Although the research was conducted within the context of COVID‐19, our findings in relation to the impact of this on women’s experiences of abortion are limited. It is likely that this was because the interviews took place at an early stage of the initial lockdown in Scotland. Further research to explore any impacts in the longer term and in different contexts is required.

### Interpretations (in light of other evidence)

Access to abortion services should be simple, transparent and appropriate for women’s needs.^26^ The telemedicine abortion service was described as affording ‘easy’ and ‘timely’ to access, with few technological or logistical issues reported. This supports research that has highlighted the potential benefits of telemedicine in improving access^27^, ^28^ and has reduced the uncertainty and anxiety that some women experience during this time.^29^

The telephone consultation was described positively by all participants; not being co‐present or seeing the clinician did not impact negatively on their experience, which supports findings from research in the USA.^30^ Notably, participants praised the caring, holistic and non‐judgemental approach of the staff during the consultation. This is an important finding because previous research suggests that the approach staff take to delivering care shapes women’s experience of abortion.^31^ Although there may be concerns about challenges in developing rapport or in conveying a caring attitude in a telephone consultation,^19^ these findings indicate that an appropriate communication style can be achieved via telephone consultations.

Although some clinicians may be concerned about the elimination of routine pre‐abortion ultrasound scanning, both the WHO and RCOG guidelines are clear that routine ultrasound is not required as part of abortion care.^26^, ^32^ In our study, most participants perceived not having a scan positively, primarily because they associated scans with the positive experience of a continuing pregnancy. Therefore, our findings contribute evidence of the acceptability of eliminating routine scanning. Nevertheless, we recognise that clinicians may be concerned that selective, rather than routine, ultrasound will mean that non‐viable pregnancies without bleeding, or rare molar pregnancies, go undetected. Findings from ongoing large robust clinical surveillance studies should help inform this aspect of the trade‐off between the former service model and the telemedicine model.^12^

Previous research has identified the importance of comfort and privacy as core features of a positive abortion experience for most women.^2^, ^19^, ^33^ Our findings highlight that the telemedicine service allows women to experience the comfort of being at home throughout the entire process: consultation, self‐administration of mifepristone and misoprostol, and passing the pregnancy. Moreover, a further benefit of telemedicine was that some participant’s reported feeling less ‘visible’ during the telephone consultation. In the context of ongoing abortion stigma, telephone consultations can help address and mitigate concerns around being seen and potentially judged by others. Nevertheless, the required privacy to conduct the consultation in the home was a challenge for some participants. This reinforces the importance of not assuming that the home is a private, safe or comfortable place for all, in which to undergo an abortion.^2^

The importance of information and support for women undergoing medical abortion at home has been highlighted.^2^, ^19^, ^25^ There were no reported issues in this study with accessing or administering abortion medications and the participants were confident about undertaking the process based on the instructions provided, with the awareness that support was available from the service if required. While it is vital to attend to issues of literacy and language competency, these findings highlight women’s competence to self‐administer both mifepristone and misoprostol at home without in‐person clinical supervision. This lends further weight to research evidence demonstrating that undergoing medical abortion at home with appropriate support, is safe and acceptable to women.^12^, ^13^, ^18^, ^19^, ^34^, ^35^

## Conclusion

This research provides evidence for the acceptability of telemedicine medical abortion at home to women. This supports the continuation of this service, beyond the temporary arrangements put in place during COVID‐19, as an option to enable those seeking abortion to access this essential health service.^36^

### Disclosure of interests

None declared. Completed disclosure of interest forms are available to view online as supporting information

### Contribution to authorship

The original idea and overall study design were conceived by JH, NB, STC and JRW. Qualitative study design, data collection and analysis were conducted by JH, NB and JRW. NB prepared the initial manuscript with contributions from, and edits by, JH, JRW and STC. All authors jointly approved the version to be published and are accountable for the accuracy and integrity of the work.

### Details of ethics approval

The qualitative study was formally reviewed and granted ethical approval by the Usher Research Ethics Group, University of Edinburgh (Application 2020; 4 May 2020). Consent for study participation was obtained at the time of interview and approved by the appropriate NHS Lothian Quality Improvement Team.

### Funding

The study was funded by the NHS Lothian Sexual Health and Blood Borne Virus Programme Fund (Ref. R46498–R46499). The study funders were not involved in research conduct or manuscript preparation. NB is supported by the Health Foundation’s grant to the University of Cambridge for The Healthcare Improvement Studies Institute. The Health Foundation is an independent charity committed to bringing about better health and health care for people in the UK. This work was carried out in part by staff at the MRC Centre for Reproductive Health (JRW and STC), which is funded by grant: MR/N022556/1.

### Acknowledgements

The authors wish to thank the women who generously agreed to take part in the study, and the clinic staff who supported recruitment to the study, in particular Anne Johnstone and Karen McCabe.

## Data Availability

The data that support the findings of this study are available from the corresponding author upon reasonable request.

## Supporting information


**Appendix S1**. Interview topic guide.

**Table S1**. NHS Lothian Medical Abortion via telemedicine consultation during COVID‐19.

Supplementary Material

Supplementary Material

Supplementary Material

Supplementary Material

**Video S1**. Author Insights: Women’s experiences of a telemedicine abortion service implemented during the coronavirus pandemic.
