# Supplementary material for: Women’s experiences of a telemedicine abortion service (up to 12 weeks) implemented during the coronavirus (COVID‐19) pandemic: a qualitative evaluation
Source: BJOG. 2021 Jul 27;128(11):1752–61. doi: 10.1111/1471-0528.16813 (PMC8441904; doi:10.1111/1471-0528.16813)
Supplement: Supplementary file 1 — Appendix S1. Interview topic guide. [file BJO-128-1752-s001.docx]

**Appendix S1.** Interview topic guide: women’s experiences of medical abortion accessed via NHS Lothian telemedicine abortion service

| **Introduction** | - Study aims - Queries, clarifications etc. |
| --- | --- |
| **General background** | - Age - Life circumstances - Area of residence, living situation |
| **Navigating access to, and expectations of, abortion service** | - Sources of information about abortion accessed prior to contacting the abortion service - How women first accessed the abortion service? (e.g. GP or self-referral etc.) - Where women accessed information on the service? (e.g. online, through GP etc.) - When and how they became aware that abortion care would be delivered by telephone consultation - Views on idea of telephone consultation for abortion - Previous experiences of teleconsultation for health care |
| **Initial contact with abortion service** | - How women chose when to call the telemedicine abortion service - What influenced the timing of their call to the service (e.g. work or household demands; space/privacy; clinic arrangements) - Preparation made by women for their initial call to the service - Experience of waiting time(s) - Time between initial call and telephone consultation |
| **Experience of the telephone consultation** | - Overall experience of consultation   - Overall experience of the consultation   - What the consultation was like for them - Benefits and drawbacks of telephone consultations   - Views on differences between in-person and telephone consults   - Preferences around telephone/video/f-t-f consultations   - Reasons for preferences for mode of consultation - Communication during the telephone consultation   - How women felt about not seeing the person they were speaking to   - How women felt about communication during the consultation   - How easy (or not) they found it to express themselves in consultation - Timing and time pressures related to telephone consultation   - Experiences of timing and time pressures; personal or from clinic - Information provision (and reception) during telephone consultation   - Information received about the abortion process during the consultation   - ‘Level’ and accessibility of explanations to prepare them for the abortion   - Amount/volume of information   - Asking questions; unanswered questions   - Accessing other sources of abortion information - Contraceptive counselling during telephone consultation   - Discussion with staff about contraception during the consultation   - Views on contraceptive counselling during teleconsultation   - Access to preferred method of contraception - Discussion of gestational stage, incl. ultrasound during telephone consultation   - How easy it was for women to identify date of last period and/or how many weeks pregnant they were at time of consult   - Confidence in providing info related to gestation   - Views on ultrasound as part of (or not) the consultation   - Feelings around not having/having a scan |
| **Accessing abortion medications** | - Process of obtaining abortion medications   - How women obtained/accessed the abortion medications pack? (e.g. collection, delivery etc.) If collected, where they collected from   - How long after the consultation did women obtain/access medications   - Views on provision of medication now, and in future - Views on contents of the medication pack   - Views on instructions contained within medication pack; clarity   - Labelling of medications in the pack   - Alignment of verbal and written instructions within medication pack |
| **Experience of abortion process and passing pregnancy at home** | - Administering the first medication (mifepristone)   - How long waited before taking the first pill (mifepristone)   - How women decided to when to take mifepristone   - Experience of taking the medication   - Feelings around taking the first medication - Administering the second medication (misoprostol)   - How long women waited between taking the first and second pills   - How women felt during this period   - Factors influenced the time chosen (e.g. supporter availability; space; work; household activities etc.)   - Preparations (if any) for taking second medication   - How women chose to administer the second pills   - What influenced decision around how to administer misoprostol - Experience of the abortion process and passing pregnancy   - Expectations and experiences of the process   - Experience of pain and bleeding   - Support from household and family members |
| **Support from abortion service** | - Pre/post treatment support from telemedicine abortion service   - Views and experiences of support available by phone to help with treatment   - Awareness of how to access support   - Accessing support from staff during the abortion process; including accessing support before or during the abortion   - Views and experiences follow up - Emotional support and post abortion counselling   - Views and experiences of provision of emotional support before and after treatment   - Experience of emotional support delivered by phone telephone   - Emotional support from family, friends and wider network |
| **Impact of COVID 19 on abortion process** | - Impact of COVID-19 on experience of treatment (beyond method of service delivery) - Impact of COVID on location of ‘home’ abortion - Impact on COVID on availability of preferred supporter - Impact of COVID on family/household presence |
| **Views on future abortion service provision** | - Views on whether the abortion service should continue to be delivered via telephone or other methods - Views on use of video versus telephone calls - Views on what, if anything, they would change about the telephone consultation |
