# Supplementary material for: Women’s experiences of a telemedicine abortion service (up to 12 weeks) implemented during the coronavirus (COVID‐19) pandemic: a qualitative evaluation
Source: BJOG. 2021 Jul 27;128(11):1752–61. doi: 10.1111/1471-0528.16813 (PMC8441904; doi:10.1111/1471-0528.16813)
Supplement: Supplementary file 2 — Table S1. NHS Lothian Medical Abortion via telemedicine consultation during COVID‐19. [file BJO-128-1752-s003.docx]

**Table S1.** NHS Lothian Medical Abortion via telemedicine consultation during COVID-19

|  | Description |
| --- | --- |
| Qualification Criteria | Pregnancy under 12 weeks gestation by last menstrual period (LMP) or ultrasound.  No signs, symptoms or significant risk factors for ectopic pregnancy. |
| Treatment pack contents | Abortion medications (mifepristone and misoprostol) and supplies of: analgesia (dihydrocodeine); anti-sickness medication (cyclizine); antibiotics (doxycycline); contraception (pills, patch, ring, condoms) and; a low‐sensitivity urine pregnancy test (LSPT) threshold 1000 iu.  Pack also contains written advice and instructions, relevant contact support numbers and an indicative plan for follow-up assessment by telephone. |
| Gestation and scanning | Ultrasound booked in clinic if: uncertain of LMP; LMP 12 or more weeks ago; inconsistent use of combined hormonal contraception, progestogen only contraception use, intrauterine device in situ; signs, symptoms or significant risk factors for ectopic pregnancy. |
| Mifepristone Administration | Self-administration of 200mg oral mifepristone at home at preferred time. |
| Misoprostol Administration | Self-administration of misoprostol (vaginal, sublingual or buccal) at home at preferred time; ideally 24-48 hours after mifepristone, but up to 72 hours, if earlier is not possible. |
| Dose of Misoprostol | Initial dose of 800 μg tablets misoprostol.  Extra dose of 400 μg misoprostol to be taken if no or minimal bleeding within 4 hours of the initial dose.  For those with gestations 10-12 weeks, a further 400 μg is provided and instructions that it should be taken if abortion has not occurred. |
| Support | Telephone support provided by the clinic during the day and hospital gynaecology out of hours (dedicated phone lines). |
